# Digitalization of relational space in the service triangle: The case study of retail banking

**DOI:** 10.3389/fsoc.2023.1141879

**Published:** 2023-03-30

**Authors:** Anna Carreri, Giorgio Gosetti, Nicoletta Masiero

**Affiliations:** ^1^Department of Human Sciences, University of Verona, Verona, Italy; ^2^School of Social Sciences, University of Hasselt, Hasselt, Belgium; ^3^Institute of Economic and Social Research (IRES) of Veneto Region, Venice, Italy

**Keywords:** digitalization, algorithm, working relationships, banking, surveillance, work identity, service triangle, quality of work

## Abstract

**Introduction:**

The article aims to shed light on the process of shaping the relational space of work in the service triangle through the progressive digitalization of work in retail banking industry. It addresses the following research question: how do technological shifts affect the relationships and interactions (a) between employees and supervisors, and (b) between employees and customers? Through a close examination of the redesign of the interpersonal relationships from the subjective viewpoint of front-line workers across these two levels, the paper contributes to advancing the understanding of the impact of technologies on surveillance practices, work identity and professional ethics in a key working sector with regard to digitalization and changes in professional requirements.

**Methods:**

The question is addressed through a qualitative case study of retail banking in Italy. In the (retail) banking sector, the redesign of the relations between supply and demand for services is more sensitive to the changes afforded by digitalization and learning algorithms. The study was conducted with the involvement of workers and trade unionists, with whom we embarked on a constant work of re-articulation through data collection, analysis, and conceptualization. We collected a multiplicity of data for triangulation: interviews, focus groups, documents, and ethnographic notes.

**Results:**

Data analysis shows how work processes and interpersonal relationships start to be redesigned across the two levels. At (a) level, two main aspects are found: the measurement of individual performance within the logic of quantification, which reduces employees to a set of measured dimensions, pushing workers into conditions of stress and competition; new surveillance practices and forms of organizational control enabled by technologies and learning algorithms. At (b) level, from being an expert with specific knowledge in the financial sector the bank employee turns into a kind of seller of any product that the algorithm decides to sell, thus ignoring the value of situated experience held by embedded, embodied social actors. Moreover, algorithms enter jurisdictional spaces traditionally controlled by knowledge workers and produce unknown outcomes concerning to whom to sell which products that cannot be clearly understood by workers.

**Discussion:**

Technology contributes to engendering complex identity constructions to maintain, protect, and revise professional identity.

## 1. Introduction

Although the ongoing digitization of work has benefited from growing attention in various disciplines, much of the debate has focused on the macro-level, counting job gains/losses (Fleming, 2019). Less has been said about whether and how the remaining professions are adapting (Anteby et al., 2016) and about the possible tensions inherent in the emerging entwining of workers and digital assemblages. We attempt to fill this gap by focusing on an under-explored dimension at the center of the technological change: the relational space of work (Sias, 2009; Heaphy et al., 2018). One of the most profound changes to the landscape of work in recent decades has involved new forms of technology, including learning algorithms, and how they have introduced the relational space of work in the so-called worker-manager-customer triangle (Korczynski, 2009, 2013; Lopez, 2010; Gabriel et al., 2015). The conceptualization of the social relations of production in the heterogeneous service sector as a triangle of employers, workers, and customers is a contribution of the sociology of service work that has become pivotal in the analytical framework of sociology of work as a whole.

Despite several calls to focus on the topic of bridging work relationships across many different communities of researchers in the last decade (Dutton and Ragins, 2007; Ferris et al., 2009; Sias, 2009), theories about the relational space of work that account for the rapid shifts in technology (Heaphy et al., 2018) are still scarce. Similarly, although the sociology of service work has produced a rich body of research, shedding light on complex and unprecedented aspects of social relations in the triangle, there has been less reflection on how technology is modifying these very relations (Lopez, 2010).

Against the backdrop of these debates, our paper is focused on the progressive digitalization of the retail banking industry and its consequences on the relational space of work in the service triangle (Korczynski, 2009, 2013; Lopez, 2010; Gabriel et al., 2015). The retail banking sector is one of those in which the redesign of the relationship between supply and demand for services is more sensitive to the changes enabled by digitalization and learning algorithms, yet scholars have paid little attention to this subset of the financial sector. One consequence of digitization is the reduction of retail bank branches and a renewal of job profiles to support the digital transformation in central offices. Little attention has been given to the transformation of work and skills among the remaining retail bank employees. In this article, we aim to portray the workplace experience as it is understood by the retail workers themselves. Importantly, traditional skills not only need to be combined with digital ones, but they must also align with the new strategic objectives of the banking institutions. In fact, technological innovation is part of a path of great change that the banking sector has undergone in recent decades, as shown in the literature. Growing managerialization, globalization and increasing competition with actors other than banks have markedly changed employment practices and the way work is traditionally performed in the banking industry: studies from different countries have shown that a process of segmentation of work organization, accompanied by the narrowing of task variation, work intensification, and Taylorization, especially for customer-facing jobs, together with increased managerial control and the strengthening of sales culture, is proven to cause worrying levels of stress, a sense of alienation and hard work identity, hence characterizing an occupation that was traditionally associated with high prestige, meaningful work and advanced technical knowledge and skills (Regini et al., 1999; McCabe, 2007, 2009; Gond et al., 2014; Kipping and Westerhuis, 2014; du Plessis and Just, 2022). Technology in this long-standing process of bank work change seems to reduce employees to the status of machines that must passively fulfill the tasks assigned to them and whose performance is measured individually through a combination of innovative control systems and older disciplinary forms of management (Regini et al., 1999; McCabe, 2007), thus opening up new challenges for trade union organizing (Murphy and Cullinane, 2021; Kornelakis et al., 2022).

Through the close examination of the subjective viewpoint of front-line bank workers on the redesign of interpersonal relationships between (a) employees and supervisors, and (b) employees and customers under the lens of “digital governance,” this article contributes to understanding the impact of digital technologies on surveillance practices, work identity conflicts, and challenges for professional ethics in a key working sector regarding digitalization and changes in professional requirements. Recently, some studies have focused on labor relations in banking (Subramanian and Suquet, 2018; Carollo and Solari, 2019; Laaser, 2019; Carollo, 2021). However, they do so without examining how relationships in the service triangle are intertwined and shaped by technologies producing specific consequences on the quality of working life (Carreri, 2020). Being aware of the difficulty of distinguishing and defining the various digital technologies as well as the types of algorithms developed so far (Dourish, 2016), we use the definition proposed by Faraj et al. of learning algorithms as an emergent family of technologies that build on machine learning, computation, and statistical techniques, as well as relying on large data sets to generate responses, classifications, or dynamic predictions that resemble those of a knowledge worker (Faraj et al., 2018, p. 62). At the same time, the analysis does not preclude other forms of digital devices that can mediate working relationships within banks. We therefore adopt a broad and composite view of the digital context in this paper.

## 2. Digitalization of relational space of work

The change in the relational space of work and its intertwining with digital assemblages and learning algorithms is a heterogeneous and ongoing phenomenon that still needs to be investigated by scholars of different disciplines. Moreover, the current debate on the increasing digitalization of the workplace is rather speculative given the scarcity of empirical research, and opinions tend to be polarized. On the one hand, there are scholars who see the potential of digital technologies to boost productivity and at the same time to improve the quality of jobs and enable social inclusion and integration (Brynjolfsson and McAfee, 2014; de Vaujany and Vaast, 2014; Autor, 2015; Barrett et al., 2015; Newell and Marabelli, 2015; Hirsch-Kreinsen, 2016). On the other hand, there are those who emphasize the risk of replacement of human labor by new technologies, the growth of inequalities, and the intensification of work for the remaining jobs (Ford, 2015; De Stefano, 2016; Frey and Osborne, 2017; Spencer, 2017; Constantinides et al., 2018; Neufeind et al., 2018; Acemoglu and Restrepo, 2020; Kellogg et al., 2020). Beyond the idea of polarization and the macro-level counting of job gains/losses, what needs to be empirically explored is how people's ways of working and the quality of work are changing due to their exposure to learning algorithms and, more generally, to digital assemblages (Carreri, 2020).

Machine learning, which is considered an extension of predictive analytics, is a method of computational learning underlying most artificial intelligence applications. Learning algorithms improve themselves without relying on explicit programming, on pre-specified instructions, but through data experience that relies on evolving networks of connections that become more refined with each additional data point (Michalski et al., 2013; Burrell, 2016). By learning from huge volumes of observations, in which data extracted in one context is combined with data from other contexts and possibly of different natures, they are capable of carrying out predictions. For example, from the economic transactions as well as the human interactions that are digitally traced, the so-called “Big Data,” along with the digital tracing of human activities through mobile devices, algorithms extract patterns that guide the actions of companies. Algorithms, by exploiting “the digitization of just about everything” (Brynjolfsson and McAfee, 2014:65), have at their disposal a much richer set of inputs with the goal of making more precise predictions.

Importantly, as with other technologies, algorithms are political in their design (Winner, 1980; Akrich, 1992), as they implicitly or explicitly include value schemes, beliefs, and ethical standards (Introna, 2016), which produce political consequences by giving certain people, objects, ideas, and events higher power and visibility than others (Ananny, 2016). These technologies have assumptions embedded within them that are not always accessible to users' understanding but can have profound effects on the knowledge created and the decisions made by using them (Faraj et al., 2018). Therefore, the ongoing technological change driven by algorithms deserves in-depth studies on the new forms of power and control that can be created.

In our comprehensive digitalized life, learning algorithms can easily do several activities that resemble those of knowledge work (Frey and Osborne, 2017; Manyika et al., 2017). Machine learning techniques can substitute workers in their routine cognitive tasks, after reducing humans' activities to sets of rules and patterns, and they can emulate the ways in which tacit knowledge is acquired by workers through long experience as well. However, it is unclear whether and how remaining professions are adapting (Anteby et al., 2016), and with what tensions inherent in the emergent entwining of workers and algorithms in dealing with managers and customers. Learning algorithms can indeed be considered performative due to the extent to which their use can shape and alter work and organizing in qualitatively different ways beyond simply signaling an acceleration of long-term technology trends (Faraj et al., 2018).

Looking at the worker-manager-customer triangle (Korczynski, 2009, 2013; Lopez, 2010; Gabriel et al., 2015), learning algorithmic outcomes appear to be more objective, neutral and reliable than humans' decisions (Boyd and Crawford, 2012). From this perspective, concerning the relationship between employees and supervisors, learning algorithmic could be used to impose a certain desirable discipline in the workplace and function as panopticons (Zuboff, 1988; Burton-Jones, 2014). For these features, they could also be seen to be less manipulable by humans with the potential result of decreasing corruption in fields such as evaluation of work performance and credit scoring in loan application processes, limiting the employee's (but also the supervisor's) discretion (O'Neil, 2016). At the same time, individuals—who are reduced to categories by algorithms—could be erroneously assigned to a certain group of people without a full understanding of how the machine made this decision, which cannot be easily explained or redressed due to the quantity and complex interaction of data fed into the algorithm. Indeed, as Dourish (2016) wrote, much of the debate about “algorithms” at the moment focuses on a particular class of algorithm—statistical machine learning techniques—which, differently from the past, produces outcomes that are unknowable in some ways even to its developers. Given the fact that learning machines are not accountable (Pasquale, 2015), the current technological change risks reducing knowledge work to an executive role and dismissing the value of the situated experience held by embedded, embodied social actors (Constantiou and Kallinikos, 2015). Knowledge workers risk having their discretionary capacity reduced and their job broken down into quantifiable sub-tasks or concrete deliverables that are subject to ratings or evaluation by supervisors but also by customers (Orlikowski and Scott, 2014; Ananny, 2016).

Concerning the relationship between employees and customers, the introduction of a strong sales culture into the service sector, including banking (Regini et al., 1999; Kipping and Westerhuis, 2014), must be taken into account. Many companies today depend on learning algorithms to better understand their customers and potential revenue opportunities to derive accurate predictions with less reliance on workers' expertise and intervention. However, research is yet to shed light on how specific contextual factors can influence relational dynamics beyond the logic of “customer sovereignty” (Korczynski and Ott, 2004), for example by reducing or reversing the power imbalance between workers and customers where employees have technical knowledge and skills. In the literature, the debates on service work and knowledge work are still poorly connected (Gabriel et al., 2015). Some studies have recently begun to move in this direction with specific reference to bank work (Carollo and Solari, 2019), however we do not know whether and how technology limits or increases the margins of agency in the relational space between workers and customers. Regarding the relationship with customers, another issue to focus on is that of ethics (Laaser, 2019). Algorithms that imitate cognition and behavior cannot grasp the ethical choices and moral dilemmas that humans deal with regularly, bringing issues of technology morality to the forefront (Verbeek, 2009; Faraj et al., 2018). This aspect appears decisive in a context such as the banking and insurance sector, marked by deep crises and difficult attributions of responsibility (Hargie et al., 2010; Bertell et al., 2020).

## 3. Materials and methods

### 3.1. The research design

The questions are addressed empirically through data collected in a large qualitative multiple case study about the impact of digital change on employees' ways of working and workplace relationships in six different working sectors in Italy. It is an action research project aimed at giving some insights into the potential activism that trade unions could perform in the face of ongoing technological and organizational changes. The project was inspired by the difficulty expressed to us by one of Italy's major trade unions in understanding current technological changes and workers' visions and needs. We involved workers and trade unionists throughout the research process to ensure concrete spillovers to all stakeholders involved.

The issue of increasing digitalization and its effects on the relational space of work inevitably raises important questions about the implementing of labor regulations and collective bargaining and, more generally, about the action of trade unions, which are now going through a transformative and challenging phase (Baccaro and Howell, 2017; Meardi, 2018; Pulignano and Doerfinger, 2018). While new forms of technology can be a constraint that limits the union's ability to represent and organize workers, on the other hand, they can be used by employees, trade unions, and other groups as tools to resist managerial action and hegemony (Jolly, 2018). Action research is suitable for addressing issues of pressing concerns by bringing together research and action, theory and practice, academic and local knowledge, and the participation of people in research (Reason and Bradbury, 2001). Action research is an emergent process of collaboration and dialogue among a team of researchers and members of an organization or a community: in our case, the research was developed by the authors and by one of the three largest trade unions in Italy. Together we formulated the problems to be examined with the purpose of generating new knowledge about these problems and identifying concrete actions to create a better situation in which members have an increased capacity to intervene.

This paper is focused on the first case study of the project concerning the digitalization of the relational space of work in the service triangle in retail banking in Italy. To address our research questions, we chose to conduct a qualitative case study (Yin, 1984) on one of the major banks in Italy, for which we use the pseudonym “Big Group.” Our fieldwork took place in the North of Italy. We collected a multiplicity of data allowing for triangulation: two explorative focus groups with employee representatives working within the banking industry; 16 semi-structured interviews with front-line bank workers (in local branches or online) employed by Big Group Bank (three of whom are also workers' representatives); one semi-structured interview with a branch manager at Big Group Bank; one qualitative interview with the trade union affairs manager at Big Group Bank; three qualitative interviews with regional union representatives of the banking sector; two talk-back focus groups bringing together some research participants (workers' representatives within the banking industry) to discuss research findings with the authors; ethnographic notes, which were taken by the first author during two trade union congresses, informal meetings with trade union representatives, and during the interviews which sometimes took place in the workplace; public documents on the banking sector in Italy, on Big Group Bank; and materials made available to us by the trade union we collaborated with.

Regarding the interviewees (see Table 1), the participants were identified with a view to have a heterogeneous sample of retail bank workers in terms of job profile, gender, age and trade union activity, as well as some participants working in key management positions at Big Group Bank. We started with the first contacts provided by the union and then we continued with a snowball method until theoretical saturation. Regarding the focus group, union representatives were recruited as key informants about the digital processes in retail banking, paying attention to the heterogeneity of the participants in terms of gender and age.

**Table 1:** List of interviewees.

**List of interviewees**			
**Front-line bank employees**			
**Age**	**Gender**	**Section**	**Local union representatives**
59	M	Foreign business	No
58	F	Family/private banking	No
57	F	Enterprise banking	No
56	M	Credit granting	Yes
55	M	Insurance section	No
55	M	Enterprises banking	No
52	F	Mortgage section	No
52	M	Family/private banking	No
39	M	Family/private banking	No
39	M	Enterprise and family banking (online branch)	Yes
37	M	Family/private banking	No
36	F	Foreign business	No
35	F	Family/private banking	Yes
35	M	Family/private banking (online branch)	No
30	M	Family/private banking (online branch)	No
48	F	Family/private banking	No
**Management job position**			
47	M	Branch manager	
56	F	Head of union affairs	
**Union representatives of the banking sector**			
46	F	Regional union representatives	

The in-person interviews lasted 90 mins to 2 h after having taken informed consent to partake in the study. During the interviews with bank workers, we asked respondents about their exposure to digital assemblages in the workplace, the main changes in their work (tasks, skills, rhythms, etc.), and their relational experiences with management, customers, colleagues and with the union.

All the data was fully transcribed, anonymized, and analyzed using software for textual analysis, Atlas.ti. A first thematic qualitative analysis (Nowell et al., 2017) was carried out by the first author. To ensure the reliability and consistency of the interpretative analytic work, the coded themes and their interrelations were then discussed among the authors, and a continuous conversation between the emerging categories and theoretical interpretations was maintained, also through the talk-back (focus groups) with union representatives.

### 3.2. The Italian banking industry and Big Group Bank

Even though Italy is a low digital density EU country, being ranked 25th on the European Commission's measure of digital development (EDPR, 2017), the banking industry is one that has been investing the most in new technologies.

The technological transformation of banks, including Big Group Bank, has significant consequences on professions. There is a strong drive to automate operations and limit the involvement of bank employees, which has led to an ongoing reduction in the number of local branches (Bonomi, 2018). The administrative work, which has increased following the transformation of channels between the bank and customers, has been moved to back-office units working remotely, while the tasks in both local and online branches have been increasingly shifted toward commercial objectives. However, the work now carried out *via* the online branch is completely different from how it was previously performed: the customer is not present in person and the interaction takes place exclusively *via* computer, telephone, and headphones. This change has caused great suffering on the part of workers who have worked at Big Group Bank the longest. Among the repercussions on job profiles, there is also the emergence of brand-new professions in support of the digital transformation: designers, knowledge engineers, and IT experts, among others.

Moreover, the digital transformation clearly affects the ways in which users interact with the bank and the world of financial services but also and equally importantly on the services that the bank is developing and offering to its customers. Some financial institutions have already started benefitting from big data analytics and learning algorithms for enhancing banking operations, like identifying patterns, analyzing connections, and addressing and resolving issues in real-time without relying on human work. Moreover, business intelligence tools can serve as instruments of power and control when they are used in the banking sector for chronological scrutiny, employees' evaluation, and their performance measurement. Yet, these new tools contribute to shaping the relationship between employees and customers. Customer Relationship Management is the most common tool used in the banking system which allows the bank to handle its customer relationships in an organized way by building a detailed record of its customers' transactions and dealings with the bank. Customer Relationship Management provides the bank with detailed information about the nature of its client's payment transactions, spending habits and their risk propensity, thus enabling the bank to offer to its customer products that are better able to meet their needs.

Big Group Bank is one of the first in Italy that has begun to move in this direction by investing a large amount of money in artificial intelligence innovations. The analysis of (big) data starts from the cross-referencing of structured internal data from payment card transactions, from financial and real estate investments, from general internal bank data, but also from external data traditionally used by banks (such as Crif and Experian), and unstructured data like posts on social media and recordings of phone calls to the Bank's call center. By using (big) data and learning algorithms, the Bank in our case study can make personalized offers to its customers and make forecasts about their consumption trends thanks to better knowledge of their risk profile and needs. Through the new technologies, the Bank can also better identify fraud through alerts on payment systems such as credit and debit cards or Automated Teller Machines (ATM). As one retail bank worker interviewed said: “The Bank forces the client to change their habits, in the sense that it puts the customer in the situation of having no other choice but to adapt to digitization and, if not, to change bank.”

Importantly, the rhetoric of the Big Group Bank, as it appears from the analysis of the public documents, is that the use of algorithms and big data is aimed at improving the quality of the service and increasing levels of customer loyalty. In the press and social media the Bank, has projected an image of itself as a bank that is shifting its main objective from sales success to the quality of the relationship with customers. Indeed, the new technology has made it possible to measure the quality of the relationship through some indicators by using analytic tools that monitor the frequency, intensity, and effectiveness of the relationship with the customer. In other words, the bank has equipped itself to know “objectively” how and how much it is taking care of its customers.

Big Group Bank is structured in four front-line segments in which employees manage different types of customers and in different ways: in one segment there are employees who have companies as customers; in another segment there are those who manage customers who have a considerable amount of capital above a certain high threshold; in another segment there are employees who deal with customers with an average (or lower) budget; finally, in the online branch there are the “employees with earphones,” as they are defined, who manage remote customers. The Bank sets different objectives for the retail employees depending on the segment to which they belong.

Finally, Big Group Bank is also particularly significant for answering our research questions because it bought some banks which went into bankruptcy, thus incorporating those workers for whom the traditional relationship of trust with customers had drastically failed.

## 4. Findings: The digital shaping of relational spaces of work in retail banking

### 4.1. The relationship between employees and supervisors: A matter of surveillance and pressure

Our analysis of the vertical relationship between retail bank employees and management shows that the evaluation of workers' individual performance by their superiors develops within an increasing logic of quantification (Espeland and Stevens, 2008), according to which workers are called to produce results that must be translated into numbers. The content of the work has changed a great deal over time: bank employees used to be evaluated on a service they had to provide to the customer who entered the branch with an explicit need, while today workers are asked to produce more and more quantifiable results, meaning the highest number of products sold (financial and non-financial alike) and of appointments obtained with new customers, particularly those defined as “lazy” from a financial point of view. There is a vivid expression commonly used by workers interviewed to define themselves that gives the idea of how this logic of quantification is changing the content of the work, the relationship with the branch manager, as well as the work identity of employees in Big Group Bank: “caged chickens.”

On average, professionalism has been very downplayed precisely because colleagues… and I am talking about those with whom I myself speak, obviously… they have the impression of being caged chickens, that is, that they have to sell what they are told must be sold there that month, regardless of customer needs, regardless of what they think of that product. (Employees' representative in the online branch, male, 37 years old. Source: explorative focus group 2).

By analyzing and combining both internal and external data, every month the marketing department of Big Group Bank publishes the so-called “production lists”—a list of customers to contact for differentiated commercial proposals for every employee. Each retail bank worker is asked to contact customers and obtain appointments to propose and sell certain products within a given period. Not only does the work intensify and the pace increase, but also the role of consultant, providing a service to the customer, almost disappears. Decisions are now made in the central offices, leaving those who work in local or online branches with commercial and more executive roles (du Plessis and Just, 2022). This model seems to have some points of continuity with Taylorfordism (Brown et al., 2011; Taska, 2017), as the expression “caged chickens” somehow underlines, and has already emerged in other ethnographic studies of bank work (McCabe, 2007).

Furthermore, by changing the content and organization of work, the evaluation system of workers' performance is also transformed. In this regard, the data collected suggests that the workers have the strong perception that their performance—also thanks to new technologies—is rigorously monitored, quantified, and measured by their superiors, as already found in other research on bank work (McCabe, 2007, 2009). In our case study the workers refer to feeling being directly monitored and somehow evaluated by the branch manager and the manager of the local area. This result tends to support the idea of new technologies as tools that function as a sort of panopticon, useful for imposing a certain discipline that takes place in precise ways in the workplace (Zuboff, 1988; Burton-Jones, 2014). However, in the case study we find a poor understanding of the underlying criteria, processes and mechanisms of performance evaluation, which often remain opaque and unclear in the eyes of retail bank workers. In the next excerpt, for example, the interviewee states that although Big Group Bank declares that performance evaluation is unrelated to sales results, the outcome of commercial campaigns can only play an important role in the evaluation.

They say that professional evaluations are not tied to quantitative performance or sales reports. […] But there is in fact a correlation […] because a proper approach to sales helps you achieve better quantitative results. […] There are these so-called production lists. (Retail bank employee, male, 37 years old. Source: interview).

Online and local Branch workers find themselves in the complicated situation of having to find a personal balance between pressure to sell coming from the management and their professional ethics, i.e., a compromise in which the production logic of the Taylor-Fordist model is reconciled with the logic of service. In the next quote, for example, a branch manager underlines how while 'production lists' are produced by algorithms on the basis of an (unknown) series of calculations, the customers to be contacted cannot be reduced to numbers and the relationships with them are built on the basis of affective and emotional components, so-called emotional labor (Hochschild, 1983), which is linked to a sense of social utility, gratification and appreciation (Bolton and Boyd, 2003). The resolution of the 'conflict' between the productive logic and that of service, which is rather complicated, falls solely on the worker, who is called to justify his actions with his superiors in the event of failure to achieve the production objectives.

The sales campaigns give you a rough idea of the latent needs, which are not expressed by the customers. On the other hand, they are created through the use of algorithms, of numbers, while customers are people, not numbers. […] And I think that those who are in sales have to find a delicate balance between what lies in the client relationship, our budget objectives, and those who are above us and closely paying attention to the completion of these lists. If sometimes you are not able to contact 100% of the people on your list, you may even have to… explain yourself. The objective of contacting these customers is to set up an appointment at the branch. It's not easy. (Retail bank employee, male, 37 years old. Source: interview).

If on the one hand the logic of quantification transforms all difference into quantity with the implications on professional ethics described above, on the other hand it also makes distinctions and ranks by assigning to each one a precise amount of something that is measurably different from, or equal to, all others (Espeland and Stevens, 2008). By so doing, individual performance and professionalism more generally risk being reduced to a matter of more or less and workers risk being placed within a competitive game logic. Data show how turning qualities into quantities creates new relationships (Espeland and Stevens, 2008). The objectives that each organizational unit must achieve, indeed, are not fixed but are constantly corrected upward by Big Group Bank, depending on the ongoing performance of all the branches. That is, on the basis of a mathematical model, the more the organizational unit gets close to achieving the goal, the more the Bank increases the goal. Taking advantage of the immediacy of data, technologies make it possible to draw up daily rankings based on the number of sales made by each organizational unit within a certain geographical area. The pressure on the employees but also on the branch manager, who is also in competition with the other managers, is therefore very strong and continual. The logic of quantification in our case study not only reduces employees to a set of measured dimensions but also pushes workers into conditions of stress and competition, as many interviewees told us. Moreover, the online channel usually used for communication between supervisors and their employees—instant messaging software—increases workers' feelings of stress and pressure as it is immediate and pervasive. This happens especially for those who work in an online branch, the so-called “employees with earphones.”

Roberto: The consequences that these sales pressures have on the health of workers. […] Stress and anxiety are the result of digitization, because it involves greater performance to achieve those results.

Luca: And also the increased closeness to the top and in any case to your hierarchical superiors.

Roberto: Constantly. […] It's not just the checking that is done sporadically, but I also say, when you receive a lot of email in a morning…

Luca: And maybe as many Lync, as many messages…

Roberto: … And saying we did not sell. Sell, sell!

Marco: You're at zero…

Roberto: You're at zero, look at the others, how much they are selling…

Marco: How come you don't? […]

Luca: I'm talking about what I see. It is a nasty chain, in the sense that: first, whoever is hounding you, probably the area manager or the director, he is probably under lots of pressure and is not living well, is not managing well and then he passes it on to you. (Explorative focus group 2 with employees' representatives).

The fact that your boss no longer has to come knocking on the door to talk to you but instead he writes a message to you, he sends you a Lync, this has made the relationship more immediate and even a little more urgent, let's say, with our managers. I see this above all in online branches, where this, that and the other arrive *via* Lync which becomes a constant pressure from the boss because probably before they didn't bother calling every five minutes on the phone like they do now with the videochat. (Employees' representative in the online branch, male, 37 years old. Source: explorative focus group 2).

The Bank evaluation of the professionalism of its workers—which tends to be resolved only in terms of sales magnitude thanks also to the new technologies' potentialities and to the abundance of data available to it—is not only a way to describe the behavior of employees but it easily turns into a surveillance practice. From our data it seems that new technologies, learning algorithms, and digital instant communication channels allow for augmenting forms of organizational control which are embedded in everyday work routines (Zuboff, 2015). The understanding of how this surveillance takes place is nevertheless opaque to the workers we interviewed (Burrell, 2016), thus increasing in them the feeling of always being observed and evaluated without knowing exactly by whom and why, and much less how to escape such a gaze, since they could be in control and govern ‘at a distance' through unknown mechanisms:

One thing that leaves me a bit puzzled about this Lync is that you can see when you are online, or rather when you are using the PC, and when you are not sitting at your desk. When you have to scan or photocopy some files, maybe you lose 20 minutes or more, and according to Lync it seems like you're not working. Then I hope that this Lync is not being checked by anyone, that there isn't someone who is there to monitor who is online and who is not, but you still wonder. So it can also be a control tool, certainly, yes. (Retail bank employee, male, 37 years old. Source: interview).

Another form of monitoring is that which comes through customer management control: any activity concerning customer management is monitored and evaluated by the company. The work of retail bank employees is broken down into quantifiable and concrete deliverables that are not only subject to ratings but also evaluated by customers (Orlikowski and Scott, 2014; Ananny, 2016) and ultimately by Big Group Bank.

Now we have a much stronger focus on customers and therefore on the return, on the feedback that the customer has on us, on the advice that has been given, on the services and products that have been given, on all the customer management follow-up after loans are granted. It is clear that technological evolution has had a great impact, especially in banks. […] So it is a very cataloged work, precise and not left at all to the creativity of the individual employee. Maybe once there was more room for maneuvering and for more decision-making in proposing the type of product. Today there are algorithms and their calculations that classify certain services should be offered to certain customers. […] We are evaluated on everything: on the results obviously and also on our behavior, that is, on the ability to use these lists, the sales ability to find the customer's interest, the ability to follow the customer in the post-sales phase. […] The whole aspect of customer care has been studied much more and has been implemented in recent years with various monitoring tools. (Retail bank employee, male, 39 years old. Source: interview).

### 4.2. The relationship between employees and customers: A matter of work identity conflicts and redemption

As shown, digitalization allows for the monitoring of all customer management activities. Besides being a surveillance practice, the monitoring—firmly tied to the logic of quantification—becomes a performance device. From being an expert with specific knowledge in the financial sector, the bank employee turns into a kind of “seller” of any product the bank, or rather the algorithm, decides to sell, risking ignoring the value of situated experience held by embedded, embodied social actors (Constantiou and Kallinikos, 2015). The retail bank employees, completely emptied of their knowledge and expertise, are reduced to mere sellers (Regini et al., 1999). If there is an agreement with the Bank, any type of shop, such as a tobacco shop, bookshop or supermarket, can perform services once reserved for the bank, such as withdrawals and payment of money. This is experienced by interviewees with a great sense of lessening of one's own value.

Let's say that the job of banking has moved more and more over the years, especially in recent years, from a service trade, that is, from a person who offers services, to a person who sells goods or products. More and more as a shopkeeper, less and less as a consultant, strictly speaking. And this also results from the fact that the bank's products are now sold in stores. For example, I want to get an estimate for changing my car, they gave me an estimate at the dealership that is better than the conditions I have as a bank employee. […] Also at the tobacco shop, the services of our bank are sold. […] So I don't even need the branch, the branch of that bank doesn't exist there, because there's no need. So if you want to survive, in the meantime you need to understand that there are shops that are starting to be banks, and you as a bank have to start being a store. (Employees' representative in the online branch, male, 37 years old. Source: explorative focus group 2).

There is also a somewhat distorted subject of work content, in the sense that the bank, which should provide a service… in reality we are only very skilled sellers! We sell. Full stop. So if you ask me: what do you do? I sell. And yes, it is a bit sad, in the sense that, at least in commerce employees have 14 paid monthly salaries, you say: well, instead I have 13, I should almost go sell socks, at least I would like it better and I would risk nothing, because here I sell and I risk too. (Retail bank employees' representative, female, 39 years old. Source: explorative focus group 1).

The algorithm—called “the machine” by employees—not only defines a target to be reached, which is continually corrected upwards as time goes by, but it also provides a list of specific products to offer to specific customers. Based on a multiplicity of data available to the Bank, the algorithm is able to “understand” customers and to make accurate predictions about their possible needs not yet made explicit, with less reliance on workers' expertise and intervention. By using algorithms, Big Group Bank improves their targeting capacities and enhances their productivity with huge consequences on the content of the work of the employees, especially for those who manage customers who have an average (or below) budget. The latter must phone a number of customers every day to propose financial and insurance products predicted by the algorithm as potentially of interest for those customers. Retail bank employees' specialized competence loses value because the algorithm takes over: “now there's a machine that does all this,” as one interviewee reported.

Luca: You feel really undervalued in your own job. This, of course, mostly affects the people who…

Marco: Are older.

Luca: …are of a certain age and have seen how things were before. […]

Martina: Anyone can do our job. It's true.

Marco: You just need to know how to sell.

Martina: You just need to know how to sell, so whether you sell shoes or you sell our product […] It's not like it used to be. […] While an adviser gave investment advice, he would analyze the market and the stocks that were sold on the market and recommend something to the client. This doesn't happen anymore. Because the bank says: you need to sell this, this, and this, which are MY products, full stop.

Roberto: The fact is that once, an analysis was performed in-depth and assumed that colleagues had a certain experience, as well as some knowledge of ratings, from their studies. They could have a degree in economics or even be a lawyer, and in that field they had developed the knowledge that allowed them to truly understand if the bank should give money to that entrepreneur or industrialist; now there's a machine that does all this! (Source: explorative focus group 2 with employees' representatives).

From this point of view, the research suggests that although there is an ongoing erosion process of retail bank employees' traditional professional identity and prestige, the relationship with customers—due to the complex nature of financial products—remains asymmetrical in favor of the front-line staff. By maintaining technical knowledge typical of knowledge work (Carollo and Solari, 2019), employees, particularly older ones, are able to build a relationship that goes beyond the logic of “customer sovereignty” (Korczynski and Ott, 2004) distinctive of the service sector, albeit with a certain difficulty that found especially in online branches. To obtain a sense of social utility and gratification, workers have to take action by bringing into play, on the one hand, their situated experience and knowledge and, on the other, some individual strategies to rebalance the needs of their two “bosses” (Troyer et al., 2000)—the management and the customer. Our analysis sheds light on retail bank workers' complicated process of redefinition of their role and their relationship with the customer due to the interaction through digital assemblages. Even if they wanted to, the employees would no longer be able to understand their bank's decision-making processes, as the learning algorithms which enter jurisdictional spaces traditionally controlled by employees (Pachidi et al., 2016) now take on an important role in banks' financial decisions and produce outcomes that cannot be easily explained by subjects (Dourish, 2016), causing a sense of alienation among them. However, the data shows that algorithms' predictions can simply be used as a “justification” for making the call, and in reality, it can end up being the employee's skill at persuasion that leads to setting up any in-person appointments with customers. It is not uncommon that while speaking to a client on the phone, other needs emerge that were unforeseen by the algorithm, which actually lead to the sale of a product. This is an example of a micro-politic through which a retail bank's employees try to create a “humanized version of the machine” (McCabe, 2007, p. 93).

So it's not impossible to reconcile customers' needs, your conscience, the branch's budget, and the demands of your supervisors. It's difficult, but not impossible. For example, a client may be contacted regarding an accident insurance policy, but during the course of the conversation, you find out that their car insurance is about to expire and perhaps that was not the objective of the sales campaign, it wasn't the reason for calling the client, the objective of the list. But it became possible to offer a service that the client needed: the car insurance. The branch benefits from this, as an extra policy is sold, and on a management level, the client was met with and a product was sold. This way all four sides can be happy. It's not impossible. (Retail bank employee, male, 37 years old. Source: interview).

Not only does the algorithm not cover all the possibilities, but it is also susceptible to failures. The emergence of failures has to do with the complexity of interactions: interactions that are not only face-to-face or face-to-screen, but that also take place within complex assemblages, contributing to the production of errors and bugs. Interestingly, the data suggest that if it is true that on the one hand the functioning of the “machine” remains obscure, on the other hand, it may also be that the very failures of the algorithm open up new margins of agency for retail bank workers, who can make use of their experience to “repair” or “prevent” some errors. For example, as one employee stated, the list provided by the algorithm recommended contacting a certain entrepreneur to offer some investments, but the bank had already had some issues in the past with this client, an entrepreneur with outstanding debts. The employee realized this and reported it to their supervisor, highlighting the mistake and deciding not to call that entrepreneur. In this case the employee's experience prevented what could have become a problem for the bank, generated by an algorithm mistake.

Carlo: They try to standardize everything. We've received some algorithm-generated lists with customers who had already gone bankrupt. One in particular had gone bankrupt twice. They spit out these lists, but then what? There are loads of offices that don't even check the lists and are convinced that there are gold mines there. […].

Interviewer: And how did the branch director explain this mistake?

Carlo: He said that they are results generated by a computer that take data from various sources, from the chamber of commerce, balance sheets, etc. But you need to see what the underlying problem is, if correct information was used, or if a company had inflated balance sheets. And in that case, it's better not to work with it. And that's where employees' experience needs to intervene. […] There are a series of things that aren't taken into account, but that a credit expert can evaluate. (Retail bank employee for companies, male, 59 years old. Source: interview).

The example shows how algorithm outputs that have economic, legal, and political consequences necessarily need to be monitored by experienced, embedded and embodied workers, so that human and technological agency become entangled. The choices that the learning machine makes can indeed be resisted by bank employees. Clearly, this example sheds light on the issue of hidden politics and the morality behind client categorization (Faraj et al., 2018). Technologies are unable to incorporate morality; they imitate cognition and behavior but cannot grasp the ethical choices that humans regularly face (Verbeek, 2009).

If you feel confident when you have a customer in front of you and you think you can offer a product based on the relationship you have built with the client, or it is the customer who asks you because he feels free to ask you, and not forced to express it, surely you have different post-sales management and a different way of doing business. I want to remind you that the banks were born as service companies eh! The massive processing of sales campaigns has led to a worsening of our work, because the customer feels pressured, under attack. [...] It is clear that the exasperated processing of sales campaigns does not lead to anything good. (Retail bank employee, male, 37 years. Source: interview).

This excerpt touches on the issue of the progressive lack of alignment with professional values. In our case study, we found the emergence of a moral conflict in retail banking between workers' traditional professional identity and the changing societal role of the Bank in the community, as well as the weakening of workers' sense of involvement and identification—it was severely compromised when the financial institutions went into crisis, with their moral hazards making headlines (Hargie et al., 2010).

## 5. Limitations and future research

Our case study faces at least three main limitations, which implicitly also identify the space for future research. First, we did not consider the managerial point of view. Although the qualitative analysis of the vertical relationship is thorough, it specifically captures the workers' view. We believe it is of great interest to explore the managerial point of view, at several levels, to fully understand the mechanisms of control and evaluation systems adopted by Big Group Bank. Moreover, the involvement of the managerial side would allow us to understand how the change brought about by the new technologies is embedded in managerial practices, and possibly in the adaptation strategies and identity-building processes of those in higher hierarchical positions.

Secondly, the other pole of the relationship with customers is also missing: we were unable to observe the experiences of customers and the local community more generally. How do customers perceive this change? How do they react to the change, what skills do they need, and how do they (de)construct their traditionally asymmetrical relationship with the bank and more specifically with the front-line staff, be it in the physical or online branch? What role does technology play in this changed relationship? Forms of further outsourcing of business processes to the customer redesign economic value chains and potentially lead to processes that are activated automatically without employee intervention. This is an important topic, but one that goes beyond our intentions and represents a space for future research. Overall, analyzing experiences from the different poles of the service triangle and not only from the side of workers in customer-facing jobs would more easily allow us to hypothesize “alternative” practices of organizing and to take into account the complexity and multiple factors at play.

Both shortcomings are related to the action research design in collaboration with the trade union, which traditionally represents workers at the lower levels of the corporate hierarchy. This represents our third limitation. We believe that action research has several strengths, but it also brings with it limitations, in particular by narrowing the sample to only certain work populations, and also to a certain extent the research questions. The interpretative keys are the result of an ongoing dialogue between researchers and trade unionists, and we believe that this exchange makes interpretation richer and able to give voice to the experiences of workers and those who represent them (Masiero, 2022). This focus has allowed us to shed light on “in-between spaces” and on individual workers' strategies of resistance and negotiation, as seen in the empirical section. Furthermore, such action research allows us to explore collective and trade union strategies in reacting to the digitalization of banking work and in exercising collective voice by potentially undertaking new battles and an alternative agenda for collective bargaining (Murphy and Cullinane, 2021; Kornelakis et al., 2022). This is a crucial and evolving issue that will be the subject of subsequent analysis and goes beyond the primary objective of this article, that is to investigate the role of new technologies in the service triangle in retail banking. At the level of interpretation, in this action research the management perspectives are underrepresented and for a more articulated understanding of the phenomenon future research needs to enlarge the sample.

This is also the reason why we are launching a second follow-up project in the same company aimed at investigating how the organizational paradigm is changing and how the changes underway produce specific repercussions in terms of the quality of work and working life (Gosetti, 2022) for the bank's various work populations that are differently located between an organizational 'center' that governs processes and a 'periphery' that performs executive tasks.

## 6. Discussion

Retail workers in a knowledge-intensive sector such as finance and banking are going through a profound change in the content of their work, which no longer conforms to the social imaginary of bank work and what they expect their work lives to be. The analysis of the case study clearly pinpoints the role of technology in this complex process of change. The worker's daily effort changes: in addition to the work becoming increasingly segmented and commercial (Regini et al., 1999; McCabe, 2007, 2009; Gond et al., 2014; Kipping and Westerhuis, 2014), it becomes even more of an executive character in the interweaving with the algorithm. Consequently, the performance evaluation system changes as well, becoming oriented toward measuring the sales of various products (not just financial ones) mostly decided by the algorithm. This system in turn, thanks to the potential of new technologies, puts the branches and retail bank employees in competition. Algorithmic management (Wood, 2021) makes it possible to not only constantly monitor and measure performance, but also to design tasks and impose a certain discipline on workers, restraining them and making them feel like “caged chickens,” especially in online branches where there is no face-to-face interaction with the customer. This work discipline establishes precise methods of carrying out tasks in a uniform manner, pre-defines the expected results (albeit then always adjusting them upwards) and establishes the rhythms of work, thus lowering the level of autonomy, which instead has traditionally guaranteed a certain social prestige to bank workers, as well as giving rise to forms of Taylorfordism (Brown et al., 2011; Taska, 2017). On the other hand, the analysis has highlighted how the criteria, processes and mechanisms underlying organizational control often remain opaque and unclear in the eyes of the workers. The introduction of new technologies helps to strengthen a competitive logic, the corporate language of game, well known in the new digital professions but much less so in the more traditional professions such as those being studied. It is a quantitative and competitive logic which does not conform with the retail bankers' sense of what makes the job meaningful. It clashes with the sense of pride and professional ethics of community service traditionally characterizing the work identity of retail bank employees. Technology therefore contributes to engendering complex identity constructions to maintain, protect, and revise professional identity (Brown, 2015).

In the analysis of the relationship with customers, this struggling identity process is evident because the monitoring, besides being a surveillance practice, becomes a performance device. The relationship with customers from the point of view of retail employees undergoes a process of standardization and depersonalization in which the re-composition of the logic of production and the logic of service falls exclusively on the shoulders of the individual. The sense of social utility and gratification are increasingly compromised and appear rather as personal achievements obtained through individual strategies of adaptation and balancing. These individual strategies involve resistance to tacit subjection to the regulative logic of competition and digital governance. Interestingly, it is not a question of “aversion” to algorithmic management, but rather of active and vigilant collaboration with digital assemblages. Human and technological agency are intertwined in different ways: first, it is the worker who feeds new data to the algorithm by recording a series of information during customer management, data that the “machine” uses for the elaboration of commercial campaigns; similarly, the worker can provide the missing information, as shown, by creating—despite the difficulties—a quality and listening relationship with the customer, for example during a phone call or an appointment at the branch, which moreover restores a sense of gratification in retail bank workers. Finally, in some cases the worker is able to correct or prevent “machine” errors, fully redeeming, so to speak, the centrality of their role in the banking organization.

However, these are individual tactics that are adopted in “in-between spaces” that are created in the intertwining of the service triangle and algorithms—“twilight areas” that may shrink or expand in the coming years; it is impossible to know what differences they will mark between physical and online branches and, importantly, between different levels of low-skilled jobs within the underside of the banking industry. In this article, we illustrated the workplace experiences as they are understood by the retail workers themselves. An important role to counterbalance the technologically induced depletion in the skill content of front-line jobs, in the autonomy of retail bank employees and in their professional identity, will be played by the capability of workers to express their collective voice through trade unions at the sectoral/local level, but also by the choices of employer associations and organizational-level strategies. Far from technological determinism, research needs to closely observe the processes of social shaping of technology and how socio-organizational forces negotiate the processes of (partial) automation of jobs.

## Data availability statement

The datasets presented in this article are not readily available because research ethics and data anonymity and confidentiality protocols do not allow access to the first level of ethnographic data as they may contain information which could run the risk of identifying the participants. Requests to access the datasets should be directed to anna.carreri@univr.it.

## Ethics statement

Ethical review and approval was not required for the study on human participants in accordance with the local legislation and institutional requirements. The patients/participants provided their written informed consent to participate in this study.

## Author contributions

AC wrote Sections 2, 3.2, 4, 5. GG wrote Section 1. NM wrote Section 3.1. Section 6 was jointly written by the authors. All authors listed have made substantial field research work and intellectual contributions. All authors approved it for publication.
